# Effects of Childhood Maltreatment on Social Cognition and Brain Functional Connectivity in Borderline Personality Disorder Patients

**DOI:** 10.3389/fpsyt.2019.00156

**Published:** 2019-03-29

**Authors:** Xochitl Duque-Alarcón, Ruth Alcalá-Lozano, Jorge J. González-Olvera, Eduardo A. Garza-Villarreal, Francisco Pellicer

**Affiliations:** ^1^Clínica de Especialidades de Neuropsiquiatría, Instituto de Seguridad y Servicios Sociales de los Trabajadores del Estado (ISSSTE), Mexico City, Mexico; ^2^Departamento de Investigación Clínica, Instituto Nacional de Psiquiatría Ramón de la Fuente Muñiz, Mexico City, Mexico; ^3^MIND Lab, Center of Functionally Integrative Neuroscience, University of Aarhus, Aarhus, Denmark; ^4^Laboratorio de Neurofisiología Integrativa, Instituto Nacional de Psiquiatría Ramón de la Fuente Muñiz, Mexico City, Mexico

**Keywords:** borderline personality disorder, social cognition, functional connectivity, brain remodeling, childhood maltreatment

## Abstract

Borderline personality disorder (BPD) is a chronic condition characterized by high levels of impulsivity, affective instability, and difficulty to establish and manage interpersonal relationships. However, little is known about its etiology and neurobiological substrates. In our study, we wanted to investigate the influence of child abuse in the psychopathology of BPD by means of social cognitive paradigms [the Movie for the Assessment of Social Cognition (MASC) and the reading the mind in the eyes test (RMET)], and resting state functional magnetic resonance imaging (rs-fMRI). For this, we recruited 33 participants, 18 BPD patients, and 15 controls. High levels of self-reported childhood maltreatment were reported by BPD patients. For the sexual abuse subdimension, there were no differences between the BPD and the control groups, but there was a negative correlation between MASC scores and total childhood maltreatment levels, as well as between physical abuse, physical negligence, and MASC. Both groups showed that the higher the level of childhood maltreatment, the lower the performance on the MASC social cognitive test. Further, in the BPD group, there was hypoconnectivity between the structures responsible for emotion regulation and social cognitive responses that have been described as part of the frontolimbic circuitry (i.e., amygdala). Differential levels of connectivity, associated with different types and levels of abuse were also observed.

## Introduction

Borderline personality disorder (BPD) is a chronic psychiatric condition characterized by high levels of impulsivity and affective instability, as well as a marked difficulty to establish and manage interpersonal relationships (1, 2). In patients with this multifactorial disorder, a genetic vulnerability has been identified (1, 2). This vulnerability may interact with environmental factors such as lower quality parental care (3, 4) and a history of child abuse, which is present in a large number of research subjects with BPD and has been proposed to be a contributing factor of the disorder (5).

Chronic difficulties in interpersonal relationships are a BPD characteristic and have been studied within the social cognition construct (6–8). With regard to performance in recognizing the emotions of others, some studies have found higher levels of performance on tests among BPD patients, including the reading the mind in the eyes test (RMET) (9, 10), while other studies have not reported any differences in the ability to infer the mental states of self and others, compared to controls (11–13). Similar results have been found in studies using a more ecological paradigm called the Movie for the Assessment of Social Cognition (MASC). It consists of a film that shows the social interaction of different characters and has been successfully used to evaluate social cognition in pathologies such as autism (14) and schizophrenia (7, 15) and correlates well with other tasks that measure theory of mind (16). In a number of studies the BPD patients showed deficits in the performance of the test compared to healthy participants, while other studies did not find this difference (15). It has been proposed that the possible deficit found in BPD is observed at the expense of a pattern of hypermentalization (17).

The neurobiological substrate of social cognition in BPD has been studied by task-related neuroimaging studies such as the RMET paradigm and stimuli adaptations that test Theory of Mind (ToM). These studies showed BPD patients have lower activation in areas within the temporal lobe, the superior and medial frontal regions, the cingulate cortex, parietal cortex, hippocampus, and the insula, as well as higher activation in bilateral amygdala, left temporal pole, medial frontal gyrus, right middle and superior temporal gyrus, left precuneus, left middle occipital gyrus, and right insula compared to controls (10, 18, 19). In addition, a lower brain response has been reported in the BPD group in the left superior temporal sulcus and gyrus in response to the modified version of the Multifaceted Empathy Test (MET) (20). Functional connectivity is described as the BOLD signal correlation between different brain regions when measured with fMRI. Most studies describe correlations observed between low-frequency fluctuations (<0.1 Hz) at resting state, which are organized in intrinsic neural networks which are the same previously described in task-related research (21–23). One of these networks is the default mode network (DMN), which shows a decreased connectivity in the precuneus (18), and the right posterior cingulate (24), as well as hyperconnectivity in the medial prefrontal cortex, the anterior cingulate cortex, and the posterior precuneus/cingulate in BPD compared to healthy subjects (25).

Most regions where differences were found in the brain function in BPD form part of the frontolimbic circuit. Dysfunction of frontolimbic circuitry is one of the most accepted models to explain the BPD symptoms, including emotional dysregulation and social cognition deficits (26). This same circuit has been related to morphologic and functional brain changes associated with a history of child abuse (27). For example, studies show a decrease in gray matter volume on the orbitofrontal cortex and temporal regions (28–30), as well as greater functional brain activation in amygdala (31) and basal ganglia in response to paradigms of emotional identification (32). Resting-state fMRI studies have shown both increased (33) and decreased (34) in functional connectivity in the fronto-amygdala circuit in samples of adolescents exposed to trauma. Another study reported a decrease in connectivity in the amygdala with the dorsal anterior cingulate cortex, precuneus, and frontal regions in adults with a history of emotional abuse (35). Overall, it may seem that the brain structures reported in BPD and child abuse are similar to those related to social cognition, which it is clinically relevant, as studies have shown that children with abuse presents deficits in social cognition (36). Due to the high prevalence of child abuse in BPD and the presence of social cognition problems in both, it seems plausible that this is an important aspect to study.

Even though brain activation has been studied regarding social cognition tasks in BPD, the relationship between functional connectivity at resting state and its association with the performance in such tasks has not been explored. The inclusion of the childhood maltreatment variable may offer information that could contribute to understanding the heterogeneity of clinical and neuroimaging results in BPD studies (37). The primary goal of this study was to assess differences compared to healthy controls in the clinical performance of social cognitive paradigms and functional connectivity in resting state and how it related to child maltreatment levels.

## Materials and Methods

For our study, we included 18 patients diagnosed with BPD and 15 controls without any psychiatric diagnosis (CN) in a cross-sectional design. Both groups were matched by age and education. Due to the higher prevalence of the psychiatric diagnosis among women, all study participants were women (38) and right-handed. Participants were recruited from the outpatient clinic of the Institute for Social Security and Services for State Workers (ISSSTE). We also recruited four participants from Instituto Nacional de Psiquiatría “Ramón de la Fuente Muñiz” from an ongoing study (39). The protocol was approved by the Ethics Committee of the ISSSTE (317.2017_P_2017) and the Ethics Committee of the Instituto Nacional de Psiquiatría “Ramón de la Fuente Muñiz.” All the participants signed an informed consent form, and the study followed the guidelines in the Declaration of Helsinki.

Patients diagnosed with BPD between 18 and 45 years old were included. The BPD diagnosis was established by the attending psychiatrist and corroborated by a psychiatrist with experience in BPD, who used the Diagnostic Interview for Borderline Revised (cut-off of 6) (40). To determine comorbidity, we used the Spanish version of the Mini International Neuropsychiatric Interview (MINI) (41). To obtain a representative sample of the clinical population, the study included patients with Major depressive disorder (MDD), posttraumatic stress disorder comorbidity (PTSD), and the use of medication. Exclusion criteria were disorder caused by use of addictive substances in the last 6 months, bipolar disorder diagnosis, schizophrenia, obsessive-compulsive disorder, eating disorders, and mental disability as described by the attending physician. For the control group, psychopathologies were ruled out with the MINI. Diagnosis of Axis II disorders was ruled out by means of SCID-II screening, and positive results were evaluated by the psychiatrist.

We measured the social cognition construct using the Spanish version (14) of Movie for the Assessment of Social Cognition (MASC) (42). This version is a 16-min video depicting social situations where the protagonists' emotions, thoughts, and social intentions are assessed through 46 multiple-choice questions. For each question, there is only one right answer. Mistakes were classified as hyper-, hypo-, and lack of mentalization. The test has high inter-rater (ICC = 0.99) and test-retest reliability (*r* = 0.97) and is highly consistent among observers (Cronbach's α = 0.86) (42). The video was provided by the author of the Spanish version (Guillermo Lahera; Universidad de Alcalá, Madrid, Spain) and professionally dubbed into Mexican Spanish with an adaptation to the Mexican accent and words. In addition, the study used the Reading the Mind in the Eyes test (RMET) to assess the ability to infer mental states with information from the eye gaze in pictures (43). Each participant was asked to choose one of four descriptions of mental states for each picture. To determine whether there was a history of childhood trauma, the Spanish version of the Childhood Trauma (self-administered) Questionnaire (CTQ) (44) was used.

### Magnetic Resonance Imaging

Imaging data were obtained using a Philips Ingenia 3 Tesla with a 32-channel phased-array head coil. We acquired structural and resting state functional (fMRI) sequences. The use of substances was ruled out during clinical interview. For the resting-state fMRI (rsfMRI), participants were instructed to remain quiet, keep their eyes open, without thinking of anything in particular and were presented with a white cross on a black background. T2^*^-weighted echo planar images were acquired with the following parameters: 36 axial slices, repetition time = 2000 ms, echo time = 30 ms, flip angle = 75°, field of view = 240 mm, slice thickness = 3.0 mm, acquisition matrix = 80 × 80, and voxel size = 3.0 × 3.0 × 3.0 mm^3^. Structural T1-weighted images were acquired with a repetition time = 7 ms, echo time = 3.5 ms, flip angle = 8°, field of view = 240 mm, slice thickness = 1.0 mm, acquisition matrix = 240 × 240, and voxel size = 1.0 × 1.0 × 1.0 mm^3^.

### Statistical Analysis of Clinical Measures

The statistical software SPSS-X version 22.0 for Windows, PC, was used for the analyses. We visually inspected the clinical data and used the Shapiro-Wilks test to assess for normality. We first compared the scores from the social cognition variables (MASC and RMET) and CTQ between the BPD and CN groups using a paired *t*-test (Mann-Whitney *U*-test for non-normal variables) with alpha of 0.05. We then performed Pearson's correlation between the MASC and RMET and CTQ scores to search for a possible relationship between childhood maltreatment and social cognition. Because patients with BPD often present depressive symptoms (45), we created a new nominal categorical variable using the MINI with the following factors: BPD with depression (*n* = 11), BPD without depression (*n* = 7), and CN. Then we used a one-way ANOVA to find differences in social cognition variables between the groups.

### Resting State Functional Connectivity Preprocessing and Analysis

Data were preprocessed and analyzed using the CONN-fMRI Functional Connectivity toolbox (46). The preprocessing pipeline prior to the analysis included: functional realignment and unwrap (subject motion estimation and correction, functional center to (0,0,0) coordinates (translation), slice-timing correction, detection of motion artifact sources with ART (Artifact Detection Tools; developed by Stanford Medicine, Center for Interdisciplinary Brain Sciences Research) (Time points exceeding the movement threshold of 2 mm or a global signal *Z*-value of nine were defined as outliners), direct segmentation and normalization (simultaneous Gray/White/CSF segmentation and normalization to MNI space), and smoothing (5 mm FWHM Gaussian filter). With a general linear model, nuisance variables were regressed out. The nuisance variables included were: subject motion parameters, raw white matter, and cerebrospinal fluid signals. To correct for physiological noise, we used the CompCor method (47). Signal time series were band-pass filtered between 0.008 and 0.09 Hz.

**Figure 1:**
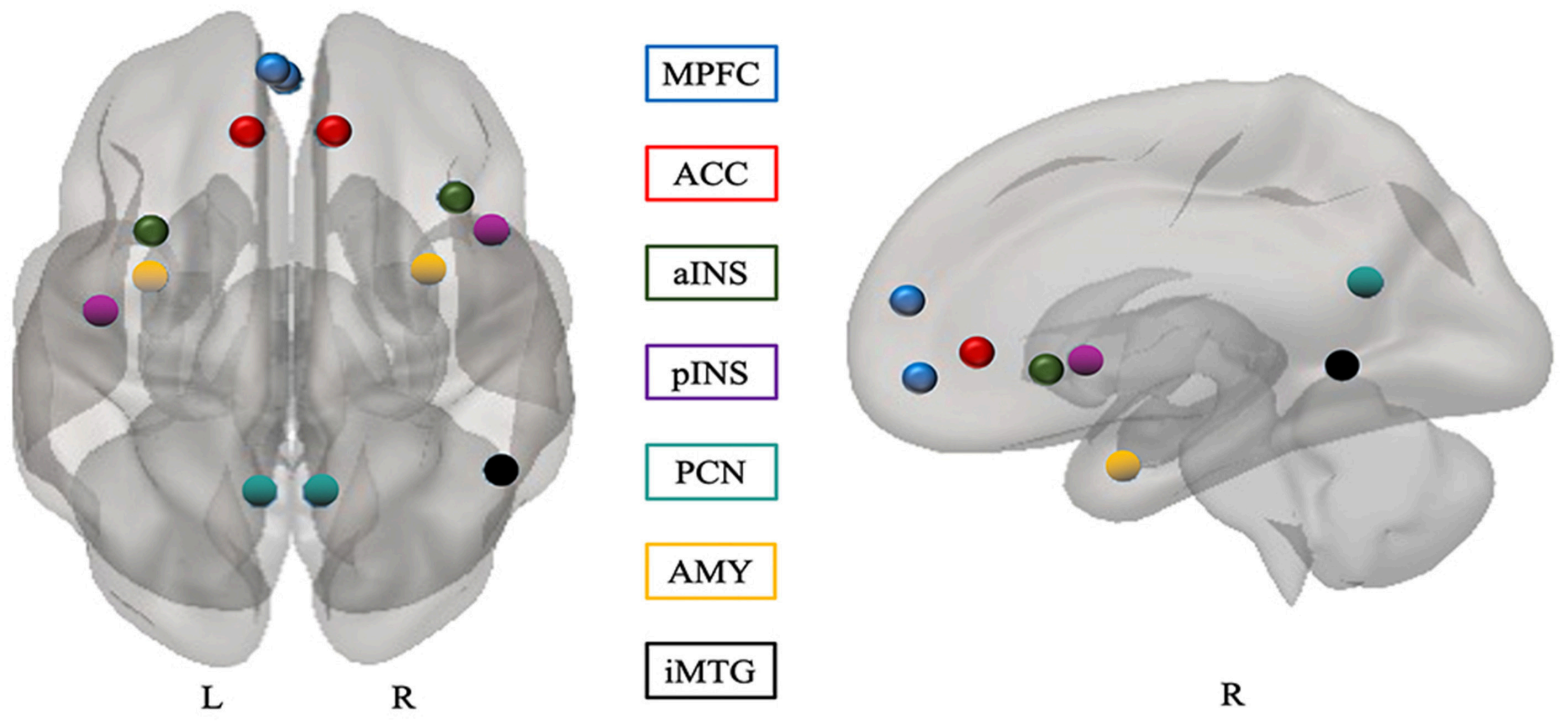
Seeds for functional connectivity. All seeds were defined based on previous studies (see Table S1 in the Supplementary Materials). MPFC, medial prefrontal cortex; ACC, anterior cingulate cortex; PCN, precuneus; iMTG, inferior middle temporal gyrus; AMY, amygdala; INS, insular cortex; L, left; R, right; a, anterior; p, posterior.

To assess baseline functional connectivity (rs-FC), we carried out a seed-based correlation analysis. The seed regions were defined in CONN using an 8 mm kernel sphere (Figure 1). The definition of the seeds was based on previous BPD results and regions associated with mentalization, especially those in the DMN (10, 18, 19, 25, 48, 49) (For details see Table S1). The whole-brain individual correlation maps were computed with the average value of the BOLD signal time course in resting state in each seed region, and correlation coefficients were estimated with the BOLD signal time course for each voxel. A normal distribution of the resulting coefficients was obtained with the Fisher transformation, and correlation maps (functional connectivity) were obtained for each seed region and subject. The correlation maps for each seed were used to carry out a second-level between-groups contrast GLM using age as a covariate. All contrasts were corrected for multiple comparisons with the false discovery rate, with a p-threshold of 0.05 for each test and cluster. Finally, we extracted the Z-maps (Fisher-transformed connectivity values) for each significant cluster in each subject to perform Pearson correlation between functional connectivity and clinical measures. Previous research indicated a higher likelihood of false positives resulting from multiple comparisons. This was particularly the case of studies that correlated brain activation with behavioral variable results, thus, we corrected for multiple comparisons (50, 51). Since depression is associated with altered functional connectivity (52) and patients with BPD often present depressive symptoms, we used a one-way ANOVA to evaluate the difference in connectivity levels associated with this variable.

## Results

### Clinical Measures

The psychiatric comorbidity and medications of the BPD group are summarized in Table S2. Compared to the controls, BPD patients showed higher scores on the CTQ. Regarding abuse subdimensions, there were no significant differences in sexual abuse between the groups (Table 1). There was a negative correlation between MASC scores and total CTQ score; for the subdimensions, there was a negative correlation between physical abuse, physical negligence, and total MASC, as shown in Table 2. In the BPD group, depression manifested in different ways. Depressed BPD subjects had lower performance on the MASC (M = 27.73, SD = 5.350) and a decrease in the mean of −5.84, 95% CI [−11.02, −0.67](*p* = 0.026), compared to non-depressed BPD subjects (M = 33.57, SD = 3.15), who even performed better than the controls on the MASC (M = 31, 17, SD = 2.57); the difference was, however, not statistically significant (*p* = 0.53), as determined by one-way ANOVA for the three groups, *F*_(2, 14.041)_ = 4.09, *p* < 0.040. No significant differences in RMET scoring were found between the groups, *F*_(2, 30)_ = 0.479, *p* = 0.305.

**Table 1:** Demographic and clinical characteristics of BPD patients and healthy participants groups.

	**BPD (*****n*** **=** **18)**		**HC (*****n*** **=** **15)**		***p*-value**
Age in years	31.17	± 9.5	32.80	±8.6	*p* = 0.613^a^
Years in education	15.06	±2.2	15.22	±2.5	*p* = 0.790^a^
CTQ TOTAL	59.06	±18.6	38.13	±7.9	*p* = 0.001^a^
Emotional abuse	16.56	±5.3	9.53	±2.4	*p* = 0.000^a^
Emotional neglect	11.44	±3.6	7.87	±3.1	*p* = 0.006^a^
Physical neglect	9.11	±3.6	6.20	±1.3	*p* = 0.006^b^
Physical abuse	11.11	±5.8	7.13	±2.2	*p* = 0.059^b^
Sexual abuse	10.83	±6.1	7.67	±4.0	*p* = 0.137^b^
MASC total correct	30.00	±5.3	31.03	±2.5	*p* = 0.449^a^
Overmentalizing errors	6.00	±2.8	5.27	±2.6	*p* = 0.538^a^
“reduced ToM” errors	5.83	±4.2	6.13	±2.7	*p* = 0.686^b^
“no ToM” errors	3.28	±2.19	3.53	±3.1	*p* = 0.714^b^
RMET	25.06	±3.26	25.73	±4.3	*p* = 0.614^a^

Data are presented in means ± standard deviation unless otherwise specified. BPD, borderline personality disorder; HC, healthy controls; CTQ, Childhood Trauma Questionnaire; MASC, Movie for the Assessment of Social Cognition; ToM, theory of mind; RMET, Reading the mind in the eyes;

a Two-sample two-tailed t-test;

b *Mann–Whitney U-test*.

**Table 2:** Table of correlations between variables of social cognition and CTQ scores.

		**1**	**2**	**3**	**4**	**5**	**6**	**7**	**8**	**9**	**10**	**11**
1	MASC total correct	1										
2	MASC overmentalizing errors	−0.02	1									
3	MASC “reduced ToM” errors	−0.77^**^^/^^++^	−0.43^*^^/^^++^	1								
4	MASC “no ToM” errors	−0.50^**^^/^^++^	−0.40^*^^/^^+^	0.48^**^^/^^++^	1							
5	RMET	0.35^*^^/^^+^	0.37^*^^/^^+^	−0.42^*^^/^^++^	−0.20	1						
6	CTQ_Total	−0.38^*^^/^^+^	−0.04	0.34^*^^/^^+^	0.09	−0.15	1					
7	Physical neglect	−0.38^*^^/^^+^	−0.05	0.42^*^^/^^++^	0.03	−0.23	0.75^**^^/^^++^	1				
8	Emotional abuse	−0.30	0.13	0.18	−0.04	−0.15	0.86^**^^/^^++^	0.59^**^^/^^++^	1			
9	Emotional neglect	−0.24	−0.34^*^^/^^+^	0.36^*^^/^^+^	0.19	−0.29	0.69^**^^/^^++^	0.62^**^^/^^++^	0.56^**^^/^^++^	1		
10	Physical abuse	−0.40^*^^/^^+^	−0.06	0.37^*^^/^^+^	0.16	−0.04	0.88^**^^/^^++^	0.59^**^^/^^++^	0.74^**^^/^^++^	0.47^**^^/^^++^	1	
11	Sexual abuse	−0.18	0.04	0.12	0.05	0.02	0.69^**^^/^^++^	0.31	0.40^**^^/^^+^	0.22	0.57^**^^/^^++^	1
	Mean	30.53	5.67	5.97	3.39	25.73	49.55	7.79	13.24	9.82	9.30	9.39
	Standard Deviation (SD)	4.31	2.74	3.59	2.63	3.38	17.99	3.18	5.56	3.81	4.92	5.49

n = 33;

** : p < 0.01 (bilateral);

* : p < 0.05 (bilateral);

+ : FDR < 0.1;

++ *: FDR < 0.05; MASC, Movie for the Assessment of Social Cognition; ToM, theory of mind; RMET, Reading the mind in the eyes; CTQ, Childhood Trauma Questionnaire*.

### Functional Connectivity

**Figure 2:**
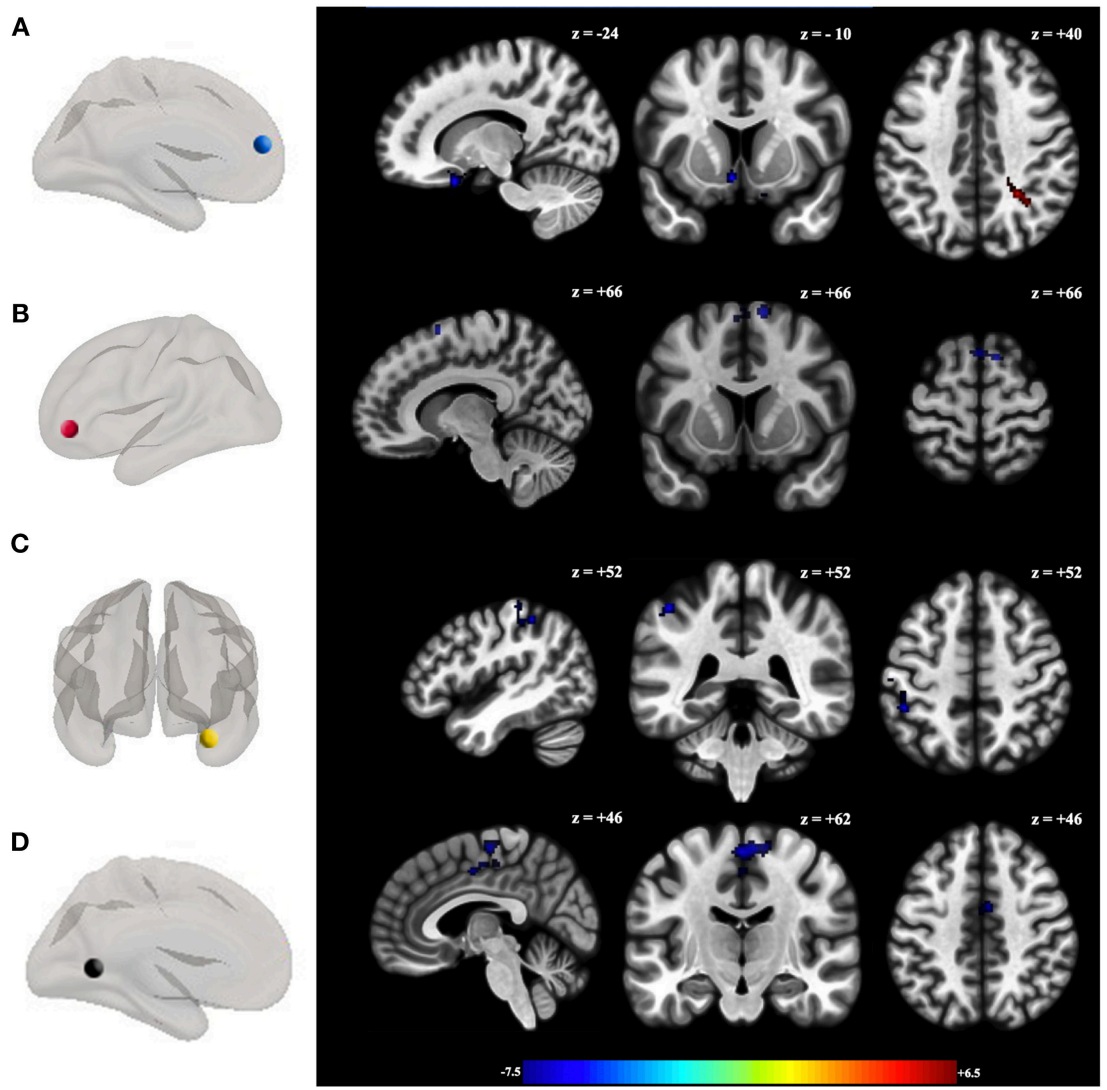
Functional connectivity differences between groups (HC > BPD). Seeds showing significant functional connectivity differences between groups with the **(A)** left medial prefrontal cortex, **(B)** anterior cingulate cortex right; **(C)** right amygdala; and **(D)** inferior middle temporal gyrus between BPD patients and healthy controls with age as covariate. All analyzed contrasts where corrected by multiple comparisons using the false discovery rate (FDR) at 0.05. Blue and orange/hot represent decreased and increased functional connectivity, respectively. The color bar indicates the *t*-value. Details of the clusters are shown in Table 3.

Hypoconnectivity was found between limbic regions that play a role in emotional and affective regulation and social responses in BPD patients. A hyperconnectivity was observed between the medial prefrontal cortex and the left superior parietal lobe (Table 3; Figure 2). There were no statistically significant differences in the connectivity values between non-depressed and depressed BPD subjects (Table S3).

**Table 3:** Seeds showing significant functional connectivity differences between groups (BPD> HC) with age as covariate.

**Seed**	**Regions**	**Peak voxel coordinate**			**Cluster size**	**β**	***t*-scores**	**Cluster significance (FDR-corrected, threshold of *p* = 0.05)**	**Connectivity**
		**x**	**y**	**z**					
MPFC_L	SPL_R	+26	−48	+40	61	0.21	6.05	0.0383	higher
	NaC.L, SubCalC, P_L, Cd_L, OFC.L	−06	+10	−10	80	−0.17	−5.82	0.0213	lower
	OFC.R, SubCalC	+14	+16	−24	76	−0.19	−7.34	0.0213	lower
ACC_R	SFG_L, SFG_R	+10	+10	+66	110	−0.18	−5.42	0.0059	lower
AMYG-R	SI_L, aSMG_L, SPG_L pSMG_L	−46	−40	+52	154	−0.16	−5.72	0.0010	lower
iMTG-R	M1_R, M1_L, SMA_R, SMA_L	+00	−16	+62	180	−0.23	−4.62	0.0008	lower
	SMA_R, M1_L, M1_L, M1_R, ACC	+04	−04	+46	131	−0.23	−4.66	0.0034	lower

*β, effect size (positive effects represent higher connectivity; negative effects represent lower connectivity); T, T-value; p-FDR, p corrected false discovery rate; L, left; R, right; a, anterior; p, posterior; i, inferior, s; superior; **Seeds**: MPFC, medial pre-frontal cortex; ACC, anterior cingulate cortex; AMYG, amygdala; MTG middle temporal gyrus. **Correlated areas**: Cd, caudate; M1, precentral gyrus (primary motor cortex); NaC, accumbens; OFC, orbitofrontal cortex; P, putamen; SI, post-central gyrus (primary somatosensory cortex); SFG, superior frontal gyrus; SubCalC, subcallosal cortex; SMG, supramarginal gyrus; SPL, superior parietal lobe; SMA, supplementary motor area. All analyzed contrasts where corrected by multiple comparisons using the false discovery rate (FDR) at 0.05. BPD, borderline personality disorder; HC, Healthy control*.

### Correlation Between Clinical and Functional Connectivity

**Figure 3:**
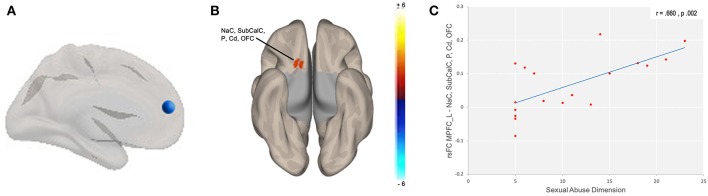
Correlation between sexual abuse and functional connectivity. Correlation between subdimension of sexual abuse and functional connectivity in **(A)** left medial prefrontal seed in BPD patients with **(B)** NaC, accumbens; Cd, caudate; OFC, orbitofrontal cortex; P, putamen; SubCalC, subcallosal Cortex; cluster: x = −06, y = +10, z = −10; **(C)** Correlation between subdimension of sexual abuse and connectivity values.

We used Fisher-transformed connectivity values of the seven clusters identified with the comparative analysis of the groups and correlated with the Movie for the Assessment of Social Cognition (MASC), Reading the Mind in the Eyes (RMET) and Childhood Trauma Questionnaire (CTQ) scores. When analyzing both groups as a homogenous group, we found that for the greater number of regions studied, a negative correlation was found between functional connectivity and the total levels of child maltreatment, as well as some subdivisions of abuse as shown in Table S4. That is, higher levels of child abuse were related to less connectivity in these regions (Figure S1). However, the above was reversed when correlation analysis was performed per group, between the CTQ and the connectivity values. In the BPD group, a negative correlation was found between the sub-dimension of emotional abuse and connectivity values in middle temporal gyrus with primary motor cortex, supplementary motor, and anterior area cingulate cortex (Cluster + 00, - 16, + 62: *r* = −0.475, *p* = 0.046; cluster +04, −04, +46: *r* = −0.496, *p* = 0.036) and positive correlation between sexual abuse score and connectivity of the left MPFC with nucleus accumbens, caudate, putamen, subcallosal cortex, and orbitofrontal cortex (cluster −06, + 10, −10: *r* = 0.660, p. 002) (Table 4; Figure 3). However, only the latter remained significant after the correction for multiple comparisons (Table S5).

**Table 4:** Correlations between functional connectivity and clinical measures in BPD patients.

**Cluster MNI**	**MPFC_L**	**MPFC_L**	**MPFC_L**	**ACC_R**	**AMYG-R**	**iMTG-R**	**iMTG-R**
Clinical Measures	*+26 −48, +40* r (*p*-value)	*−06+10 −10* r (*p*-value)	*+14+16 −24* r (*p*-value)	*+10 +10 +66* r (*p*-value)	*−46 −40 +52* r (*p*-value)	*+00 −16 +62* r (*p*-value)	*+04 −04 +46* r (*p*-value)
CTQ TOTAL	−0.003 (0.991)	0.338 (0.171)	0.149 (0.554)	−0.065 (0.797)	0.200 (0.427)	−0.107 (0.674)	−0.017 (0.946)
Emotional abuse	0.088 (0.729)	0.275 (0.269)	0.176 (0.485)	−0.094 (0.710)	0.186 (0.461)	−0.261(0.296)	−0.169 (0.502)
Emotional neglect	−0.168 (0.505)	−0.164 (0.516)	−0.405 (0.096)	0.121 (0.633)	0.227 (0.364)	**−0.475 (0.046)**	**−0.496 (0.036)**
Physical neglect	0.250 (0.317)	−0.057 (0.823)	−0.129 (0.610)	−0.034 (0.893)	0.177 (0.483)	0.015 (0.954)	0.103 (0.684)
Physical abuse	−0.112 (0.659)	0.255 (0.307)	0.226 (0.368)	−0.085 (0.737)	0.282 (0.257)	0.018 (0.945)	−0.036 (0.885)
Sexual abuse	−0.027 (0.914)	**0.669 (0.002)^+^**	0.400 (0.100)	−0.087 (0.733)	−0.061 (0.809)	0.156 (0.536)	0.358 (0.144)

*MPFC, medial prefrontal cortex; ACC, anterior cingulate cortex; AMYG, amygdala; MTG middle temporal gyrus; L, left; R, right: Numbers represents: Pearson coefficient, (p-values); Bold indicates P < 0.05 (unadjusted for multiple comparisons); MASC, Movie for the Assessment of Social Cognition; ToM, theory of mind; RMET, Reading the mind in the eyes; CTQ, Childhood Trauma Questionnaire*.

+ *: FDR < 0.05*.

## Discussion

We found that as the level of childhood maltreatment increased, the performance on the MASC social cognitive test decreased in both the BPD and the control groups. In addition, we observed hypoconnectivity between structures associated with emotion regulation and structures associated with social cognitive responses in the BPD group.

Our findings are consistent with those of another study, where they showed that child abuse impacts the skills that are necessary for the development of stable and long-lasting interpersonal relationships (36). It has been well-established that the development of social cognition is linked to that of emotional and affective communication through primary caregivers, and an environment that is safe and free from excessive stress-conditions that do not exist in the case of child abuse (53). Several studies have demonstrated that either disruptions in the relationships between children and primary figures or extremely stressful environments can activate the hypothalamic-hypophyseal axis, releasing, and activating several mechanisms that have an effect on the brain (54, 55). Environments characterized by extreme stress cause physiological changes that interfere with the integration of mental representations during development, thus disrupting the concepts of self and other and producing an unrealistic, unstable, and disproportionate representation of the affection perceived and expressed (3). These circumstances arise in relational contexts, a fact that accounts for BPD patients' clinical characteristics. These traits include aggression, impulsivity, and dissociative symptoms that are observed in BPD patients.

We did not find differences between social cognitive tasks for both paradigms, which contrast with the ongoing controversy regarding a BPD patient's ability to read mental states. Data collected with the MASC goes beyond the underlying process of recognizing emotions during social interactions by collecting data from the eye gaze, as evaluated by RMET (43). It also includes an assessment of the content of the mental state of the “other,” based on contextual information and elements that are not physically evident (16). This suggests that the instrument is ideal as it reflects real-life situations. Nevertheless, the two paradigms evaluate the cognitive dimension of the social cognitive process, and it is impossible to rule out the limitations of the affective dimension that are associated with emotional regulation and the difficulties in distinguishing between self and the other in BPD subjects (56). Our study showed that depression is associated with decreased social cognitive performance. Although other research has found similar results (57), not all studies have found differences between BPD and control groups, and other researchers have surmised that social cognition performance is a trait rather than a state of mind (58).

### Functional Connectivity Results

Our results identified differences in the organization of brain connectivity patterns between the groups, primarily finding hypoconnectivity between the regions explored. It noteworthy that these regions are components of the fronto-limbic circuit and are related to a broad range of cognitive and emotional processes, which have been described as being altered in BPD. These processes have been studied as dysfunctional traits in this disorder and include emotional dysregulation, impulsiveness, aggressive behavior, suicidal tendencies, and self-destructive behavior (59). Some of these regions are a part the default mode network, which is usually related to processes that involve thought formation by self as autobiographical memory, future planning, and inferring one's mental states and those of others (60), which are all processes involved in social cognition (61). The MPFC is involved in the detection of emotions and the process of self-other differentiation (62, 63), while the ACC regulates the monitoring of behavior, the detection and prediction of error, decision making, and processes related to self-evaluation in social contexts (64, 65). On the other hand, the temporal lobe participates in the processing of language and facial expressions, which are two essential elements in the theory of the mind and mentalization (66, 67). Finally, the amygdala has a critical role in the emotional aspect of the interactional experience (68).

Some of the results observed in our study coincide with those of multiple previous studies that examined the resting state connectivity, such as the findings for the temporal lobe, which represents one of the most commonly replicated results among this group of patients (25, 48, 69). However, with regards to the MPFC and the ACC, some studies have also found a decrease in connectivity (70, 71) while others have found increased connectivity (25, 72). The results observed in the amygdala are similar to certain regions of brain activation observed in response to emotional processing tasks (73), although the result we obtained as the opposite to what was observed in resting state, where an increase in connectivity has been reported (72). This may be explained by the effect of medication on the connectivity in this structure or by the different preprocessing methodologies between studies (74). The effect of medication should be investigated with treatment-naïve patients, while the latter is still an open debate on which is the appropriate preprocessing pipeline (75). Although differences in brain connectivity in regions that are considered to be strongly associated with social behavior were observed in this study, we did not observe any correlations between connectivity and the clinical variables of social cognition. Differences in the abilities of BPD patients to assess mental states have been observed previously, especially under conditions of emotional stress (76, 77). In that sense, we assume that the clinical performance in social cognition tasks related to brain organization at rest could vary under intense emotional states. Stress is associated with an abnormal pattern of deactivation of intrinsic neural networks (78, 79), which could be associated with variations in the performance of social skills. However, it was not possible to show the effects of stress on brain organization in this study.

When we studied both groups together, we observed an effect of child maltreatment on connectivity. Higher levels of child abuse were associated with reduced levels of brain connectivity. However, this result disappears when analyzing per group. The group analysis showed that different types of abuse had differential effects on the connectivity values in the BPD group. The experience of emotional neglect was associated with lower connectivity for the temporal lobe, although this result should be viewed with caution because it did not survive the multiple comparisons correction. In addition, a strong positive correlation was observed between the sub-dimension of sexual abuse and connectivity in the reward circuit (80). Studies have associated hyperconnectivity in this circuit with the presence of psychotic symptoms (81). This finding may explain the tendency toward excessive mental state attribution (over mentalizing) in BPD patients (82), although, in our study we did not find more overmentalizing errors in BPD patients than in controls.

It has been proposed that child maltreatment can be studied as an “ecophenotype,” a phenotype modified by specific adaptive response to environmental factors, in order to disentangle heterogeneous diagnoses such as BPD (37). Our results strengthen this approach even though it seems necessary to take into account not only the levels of abuse, but also the type of abuse experienced. In addition, it would be important to study the presence of vulnerability factors associated with BPD, since in the case of the control group, no effect of abuse on connectivity was observed. Another explanation for our result may be an underpowered analysis.

Although the effect of child maltreatment on brain structures has been widely documented (32, 83, 84), the mechanism with which child abuse might have an impact on organization and functional connectivity is still unclear. A possible explanation may be that child abuse is a factor associated with brain remodeling rather than a harmful factor *per se* (37, 85), especially within corticolimbic structures, as shown by a preclinical study of adolescent rats (86). This “modeling” effect on brain organization is present especially during critical stages and processes, such as pruning, that is necessary for normal brain development (87). In the first 2 years of life, a synaptic overproduction occurs in the brain, followed by remodeling through pruning, and these processes continue into adolescence (88). Although remodeling occurs due to cellular programming, researchers have reported that pruning in this second phase is highly sensitive to experience (89), including stress, due to the effect of inflammation mechanisms on glial cells (90, 91). This seems consistent with the new paradigm that regards the brain as an active system that self-organizes dynamically, based on the information that it receives (92). This paradigm has been studied in schizophrenic and autistic patients, for whom dysfunctional pruning has been proposed (88, 93). In BPD patients, stressful childhood events or a lack of proper parenting, may be associated with remodeling and differential pruning. Such hypothesis needs to be tested, as it would enhance our understanding of BPD as a result of maladaptive brain remodeling, resulting from the effects of traumatic experiences on brain development.

Finally, there are some limitations in our study as the lack of differences in the MASC and RMET scores between groups, and significant differences in childhood maltreatment levels. Nevertheless, even with these similarities in the MASC and RMET scores, we found differences in brain connectivity suggesting a dysfunctional resting state that should be explored further.

## Conclusion

BPD patients endured more child abuse than controls, which correlated with poorer performance on the MASC social cognitive test. We showed that BPD was associated with altered functional connectivity between structures involved in emotion regulation and social cognitive responses that are part of the frontolimbic circuitry. The rsfMRI results provide information about resting state networks that seem altered in BPD patients, and that were associated with different types and levels of abuse. Further investigation of these results is necessary to determine whether the introduction of safeguards to avoid abuse and stress during critical periods, such as childhood and adolescence, would be beneficial, and whether patients can recover from these harmful effects.

## Data Availability

The datasets generated for this study are available on request to the corresponding author.

## Author Contributions

XD-A, FP, EG-V, and JG-O were involved in the design of the research protocol. XD-A, RA-L, and EG-V contributed to acquisition and analysis of data; XD-A, FP, and EG-V drafted the manuscript, and all authors contributed revising and approved it for publication.

### Conflict of Interest Statement

The authors declare that the research was conducted in the absence of any commercial or financial relationships that could be construed as a potential conflict of interest.
