# Supplementary material for: Effects of Childhood Maltreatment on Social Cognition and Brain Functional Connectivity in Borderline Personality Disorder Patients
Source: Front Psychiatry. 2019 Mar 29;10:156. doi: 10.3389/fpsyt.2019.00156 (PMC6452291; doi:10.3389/fpsyt.2019.00156)
Supplement: Supplementary file 1 [file Data_Sheet_1.docx]

Supplementary Material

Effects of childhood maltreatment on social cognition and brain functional connectivity in borderline personality disorder patients.

Xochitl Duque^1^, Ruth Alcalá-Lozano ^2^, Jorge J. González-Olvera^2^, Eduardo A. Garza-Villarreal^2,3 *^,Francisco Pellicer ^4 *^

*** Correspondence:**

Francisco Pellicer

pellicer@imp.edu.mx

Eduardo A. Garza-Villarreal

egarza@imp.edu.mx

| Table S1. Details on the functional seeds of interest. Coordinates MNI (x,y,z) | | | | | |
| --- | --- | --- | --- | --- | --- |
| **Seed** |  | ***x*** | ***y*** | ***z*** | References |
| MPFC |  | -1 | 49 | -5 | (1) |
| MPFC_L | L | -4 | 52 | 16 | (1) |
| ACC | R | 10 | 35 | 2 | (2) |
|  | L | -10 | 35 | 2 | (2) |
| PCN | R | 7 | -60 | 21 | (2) |
|  | L | -7 | -60 | 21 | (2) |
| iMTG | R | 50 | -54 | 2 | (1) |
| AMYG | R | 33 | -1 | -29 | (3) |
|  | L | -33 | -3 | -18 | (4) |
| aINS | R | 46 | −66 | 16 | (5) |
|  | L | -33 | 9 | 6 | (5) |
| pINS | R | 48 | 9 | 0 | (5) |
|  | L | -45 | -12 | 3 | (5) |
| **Seeds**: MPFC, medial prefrontal cortex; ACC, anterior cingulate cortex; PCN, precuneus; iMTG inferior middle temporal gyrus; AMYG, amygdala; INS, insular cortex; a, anterior, p, posterior; R right hemisphere; L left hemisphere: | | | | | |

| Table S2. Psychiatric comorbidity, and medication | | |
| --- | --- | --- |
|  | BPD (n= 18) | |
|  | n | % |
| Disorders, n (%) |  |  |
| Current MDD | 11 | 61.11 |
| Past MDD | 18 | 100 |
| Social phobia | 1 | 5.5 |
| Specific phobia | 1 | 5.5 |
| Current panic disorder | 1 | 5.5 |
| Past panic disorder | 1 | 5.5 |
| Generalized anxiety disorder | 1 | 5.5 |
| Current PTSD | 1 | 5..5 |
| Medication, n (%) |  |  |
| Without medication | 1 | 5.5 |
| ATD | 9 | 50 |
| ATD/AAP | 3 | 16.6 |
| ATD/ AAP/AED | 2 | 11.1 |
| ATD/AAP/BZD | 2 | 11.1 |
| ATD/AAP/ AED/BZD | 1 | 5.5 |
| BPD, borderline personality disorder; MDD, major depressive disorder; PTSD, post-traumatic stress disorder; ATD, antidepressants; AAP, atypical antipsychotic; AED, anti-epileptic drug; BZD, benzodiazepines | | |

| Table S3. Mean connectivity values and depression (Results of one-way ANOVA, Bonferroni post hoc test) | | | | | | | | | | |
| --- | --- | --- | --- | --- | --- | --- | --- | --- | --- | --- |
|  | | | | One-way ANOVA | | | Post-hoc comparison | | | |
|  | | | | HC  (n = 15) | BPD  (n = 7) | BPD-MDD  (n = 11) |  | HC vs. BPD | HC vs.  BPD-MDD | BPD vs. BPD-MDD |
|  | cluster | | | M+ SD | M+SD | M+SD | F | p | p | p |
| MPFC_L | +26 | -48 | +40 | -0.2137+0.088 | 0.0094+0.133 | -0.0153+ 0.089 | 17.961*** | p < .01 | p < .01 | ns |
|  | -06 | +10 | -10 | 0.2392+0.080 | 0.0663+0.062 | 0.0671+ 0.099 | 17.172 *** | p < .01 | p < .01 | ns |
|  | +14 | +16 | -24 | 0.1872 + 0.078 | 0.0091+0.084 | -0.0091+0.056 | 27.327*** | p < .01 | p < .01 | ns |
| ACC_R | +10 | +10 | +66 | 0.1854 +0.071 | 0.0533+0.110 | -0.0273+0.107 | 17.068*** | p < .01 | 0.012 | ns |
| AMYG-R | -46 | -40 | +52 | 0.1181+0.092 | -0.0622+0.059 | -0.0225+0.058 | 17.712*** | p < .01 | p < .01 | ns |
| iMTG-R | +00 | -16 | +62 | 0.2348+0.162 | 0.0015+0.112 | 0.0043+0.129 | 10.713*** | p < .01 | p < .01 | ns |
|  | +04 | -04 | +46 | 0.2570+0.148 | 0.0392+0.093 | 0.0203+0.149 | 11.118*** | p < .01 | p < .01 | ns |
| HC = healthy control.; BPD, borderline personality disorder; BPD-MDD, borderline personality disorder and depression; MPFC, medial prefrontal cortex; ACC, anterior cingulate cortex; iMTG, inferior middle temporal gyrus; AMY, amygdala; L, left; R, right; ns: not significant; ***: p-value <0.01. | | | | | | | | | | |

| Table S4. Correlations between functional connectivity and clinical measures | | | | | | | |
| --- | --- | --- | --- | --- | --- | --- | --- |
| Cluster | **MPFC_L** | **MPFC_L** | **MPFC_L** | **ACC_R** | **AMYG-R** | **iMTG-R** | **iMTG-R** |
| MNI  Clinical Measures | *+26 -48,+40*  r (p value) | *-06+10 -10*  r (p value) | *+14+16 -24*  r (p value) | *+10 +10 +66*  r (p value) | *-46 -40 +52*  r (p value) | *+00 -16 +62*  r (p value) | *+04 -04 +46*  r (p value) |
| MASC total correct | -0.135 (0.452) | 0.220 (0.218) | 0.201 (0.261) | 0.355 (0.042) | -0.050 (0.779) | 0.095 (0.597) | 0.1263 (0.483) |
| Overmentalizing errors | 0.011 (0.951) | -0.146 (0.414) | -0.139 (0.437) | -0.248 (0.164) | -0.210 (0.240) | -0.092 (0.607) | -0.048 (0.790) |
| “reduced ToM” errors | 0.062 (0.729) | -0.068 (0.707) | -0.090 (0.617) | -0.130 (0.470) | 0.139 (0.438) | 0.030 (0.864) | 0.030 (0.865) |
| “no ToM” errors | -0.072 (0.688) | -0.143 (0.426) | -0.181 (0.312) | -0.081 (0.652) | 0.142 (0.430) | 0.032 (0.856) | -0.161 (0.370) |
| RMET | -0.362 (0.038) | 0.212 (0.234) | 0.272 (0.124) | 0.242 (0.174) | 0.031 (0.861) | -0.078 (0.663) | -0.064 (0.721) |
| CTQ TOTAL | **0.443 (0.009)**^+^ | -0.248 (0.162) | **-0.411(0.017)**^+^ | **-0.427(0.013)**^+^ | -0.389 (0.025) | **-0.409 (0.018)**^+^ | **-0.395 (0.022)** |
| Emotional abuse | **0.480 (0.004)**^+^ | **-0.393 (0.023)** | **-0.431 0.012)**^+^ | **-0.503 (0.002)**^+^ | **-0.495 (0.003)**^+^ | **-0.508 (0.002)**^+^ | **-0.483 (0.023)** |
| Emotional neglect | 0.309 (0.079) | -0.330 (0.060) | **-0.471 (0.005)** ^+^ | -0.231 (0.194) | -0.288 (0.102) | **-0.427 (0.013)** ^+^ | **-0.437 (0.004)**^+^ |
| Physical neglect | **0.469 (0.005)** ^+^ | -0.323 (0.066) | **-0.426 (0.013)**^+^ | -0.340 (0.052) | -0.291 (0.099) | -0.236 (0.186) | -0.176 (0.326) |
| Physical abuse | 0.234 (0.188) | -0.142 (0.430) | -0.233 (0.191) | -0.335 (0.056) | -0.227 (0.203) | -0.211 (0.237) | -0.258 (0.145) |
| Sexual abuse | 0.267 (0.132) | 0.127 (0.480) | -0.127 (0.480) | -0.232 (0.193) | -0.199 (0.266) | -0.203 (0.256) | -0.168 (0.349) |
| MPFC, medial pre-frontal cortex; ACC, anterior cingulate cortex; AMYG, amygdala; MTG, middle temporal gyrus; L, left; R, right; Numbers represents: Pearson coefficient, (p values); MASC, Movie for the Assessment of Social Cognition; ToM, theory of mind; RMET, Reading the mind in the eyes; CTQ, Childhood Trauma Questionnaire. +: FDR < 0.1; No significant values ​​were observed after FDR.05 | | | | | | | |

**Figure S1.** Correlation between childhood trauma (total CTQ score) and functional connectivity.


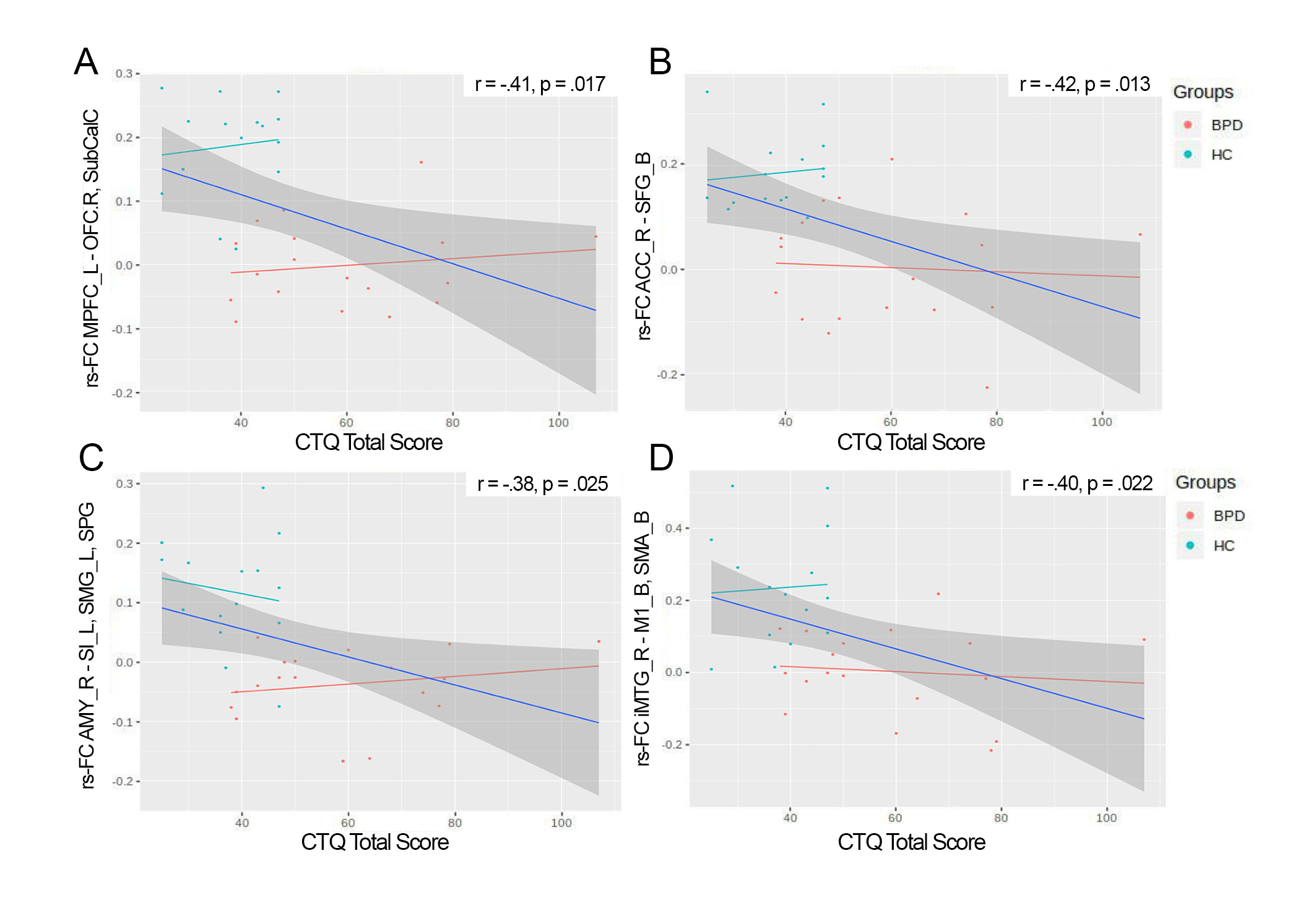


Fig 1s Correlation between childhood trauma (total CTQ score) and functional connectivity. The figure shows connectivity values for seeds and cluster: (A) MPFC_L, left medial prefrontal cortex, cluster (x = +14, y = +16, z = -24); (B) ACC_R, right anterior cingulate cortex, cluster (x = +10, y = +10, z = 66) : (C) AMY_R, right amygdala, cluster (x = -46, y = -40, z = +52) and (D) iMTG_R, inferior Middle temporal gyrus (x = +00, y = -16, z = +62). rs- = correlation coefficient. BPD = borderline personality disorder, HC = healthy control.

| Table S5. Correlations between functional connectivity and childhood maltreatment in HC | | | | | | | |
| --- | --- | --- | --- | --- | --- | --- | --- |
| Cluster MNI | **MPFC_L** | **MPFC_L** | **MPFC_L** | **ACC_R** | **AMYG-R** | **iMTG-R** | **iMTG-R** |
| Clinical Measures | *+26 -48,+40*  r (p value) | *-06+10 -10*  r (p value) | *+14+16 -24*  r (p value) | *+10 +10 +66*  r (p value) | *-46 -40 +52*  r (p value) | *+00 -16 +62*  r (p value) | *+04 -04 +46*  r (p value) |
| CTQ TOTAL | 0.098 (0.728) | 0.377 (0.166) | 0.111 **(**0.694) | 0.110 **(**0.695) | -0.150 (0.594) | 0.053 **(**0.852**)** | -0.035**(**0.901**)** |
| Emotional Abuse | -0.343 (0.211) | -0.068 **(**0.810) | 0.406 **(**0.134) | -0.022 **(**0.939**)** | -0.438 **(**0.102**)** | 0.040**(**0.888**)** | 0.066**(**0.816**)** |
| Emotional neglect | 0.104 (0.713) | 0.311 (0.259) | 0.105 **(**0.710) | 0.234 (0.401) | -0.031 (0.913) | 0.121 **(**0.667**)** | 0.173 **(**0.537**)** |
| Physical neglect | 0.137 (0.627) | 0.310 (0.260) | -0.083 **(**0.767) | -0.012 (0.966) | -0.104 (0.712) | 0.336 (0.220) | 0.508 (0.053) |
| Physical abuse | -0.111 (0.694) | 0.315 (0.253) | 0.111 (0.695) | -0.055 (0.845) | -0.177 (0.528) | 0.249 (0.371) | 0.140 (0.618) |
| Sexual abuse | 0.325 (0.237) | 0.256 (0.356) | -0.131 (0.641) | 0.083 (0.768) | 0.118 (0.676) | -0.265 (0.339) | -0.489 (0.064) |
| MPFC, medial prefrontal cortex; ACC, anterior cingulate cortex; AMYG, amygdala; MTG Middle temporal gyrus; L, left; R, right Numbers represents: Pearson coefficient, p values; MASC, Movie for the Assessment of Social Cognition; ToM, theory of mind; RMET, Reading the mind in the eyes; CTQ, Childhood Trauma Questionnaire. +: FDR < 0.1; No significant values ​​were observed after FDR.05 | | | | | | | |

**References**

1. Visintin E, Pan C De, Amore M, Balestrieri M, Christian R, Sambataro F. Mapping the brain correlates of borderline personality disorder : A functional neuroimaging meta-analysis of resting state studies. (2016) **204**:262–269. doi:10.1016/j.jad.2016.07.025

2. O’Neill A, D’Souza A, Samson AC, Carballedo A, Kerskens C, Frodl T. Dysregulation between emotion and theory of mind networks in borderline personality disorder. *Psychiatry Res - Neuroimaging* (2015) **231**:25–32. doi:10.1016/j.pscychresns.2014.11.002

3. Frick C, Lang S, Kotchoubey B, Sieswerda S, Dinu-Biringer R, Berger M, Veser S, Essig M, Barnow S. Hypersensitivity in borderline personality disorder during mindreading. *PLoS One* (2012) **7**: doi:10.1371/journal.pone.0041650

4. Mier D, Lis S, Esslinger C, Sauer C, Hagenhoff M, Ulferts J, Gallhofer B, Kirsch P. Neuronal correlates of social cognition in borderline personality disorder. *Soc Cogn Affect Neurosci* (2013) **8**:531–537. doi:10.1093/scan/nss028

5. Doll A, Sorg C, Manoliu A, Wöller A, Meng C, Förstl H, Zimmer C, Wohlschläger AM, Riedl V. Shifted intrinsic connectivity of central executive and salience network in borderline personality disorder. *Front Hum Neurosci* (2013) **7**:727. doi:10.3389/fnhum.2013.00727

**
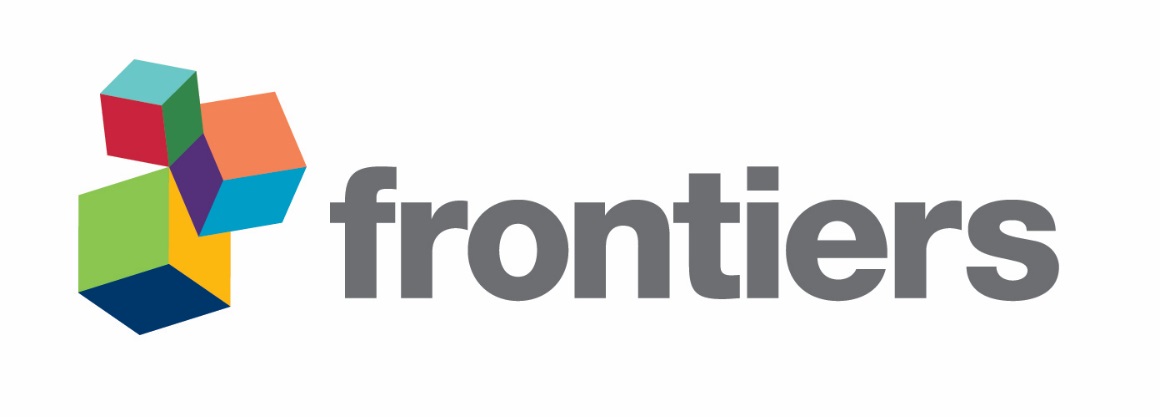
**
